# Correction: Althenayyan et al. Alternatively Spliced Isoforms of *MUC4* and *ADAM12* as Biomarkers for Colorectal Cancer Metastasis. *J. Pers. Med.* 2023, *13*, 135

**DOI:** 10.3390/jpm16010057

**Published:** 2026-01-22

**Authors:** Saleh Althenayyan, Mohammed H. AlMuhanna, Abdulkareem AlAbdulrahman, Bandar Alghanem, Suliman A. Alsagaby, Abdulaziz Alfahed, Glowi Alasiri, Mohammad Azhar Aziz

**Affiliations:** 1 Colorectal Cancer Research Program, Department of Cellular Therapy and Cancer Research, King Abdullah International Medical Research Center, Riyadh 11481, Saudi Arabia; 2 King Saud Bin Abdulaziz University for Health Sciences, Riyadh 11481, Saudi Arabia; 3 Department of Medical Genomics, King Abdullah International Medical Research Center, Riyadh 11481, Saudi Arabia; 4 Department of Core Facilities and Platforms, King Abdullah International Medical Research Center, Riyadh 11481, Saudi Arabia; 5 Department of Medical Laboratory Sciences, College of Applied Medical Sciences, Majmaah University, Majmaah 11932, Saudi Arabia; 6 Department of Medical Laboratory Sciences, College of Applied Medical Sciences, Prince Sattam bin Abdulaziz University, Alkharj 11942, Saudi Arabia; 7 Department of Biochemistry, College of Medicine, Al Imam Mohammad Ibn Saud Islamic University (IMSIU), Riyadh 13317, Saudi Arabia; 8 Interdisciplinary Nanotechnology Center, Aligarh Muslim University, Aligarh 202002, India

**Error in Figure 3**

In the original publication [1], Figure 3B contained an incorrect schematic that did not represent *ADAM12* alternative splicing as described in the manuscript. The figure legend is correct; the error was limited to the figure itself. The corrected Figure 3 (Alternative splicing of *ADAM12* protein-coding isoforms) appears below.

The authors state that the scientific conclusions are unaffected. This correction was approved by the Academic Editor. The original publication has also been updated.

## Figures and Tables

**Figure 3:**
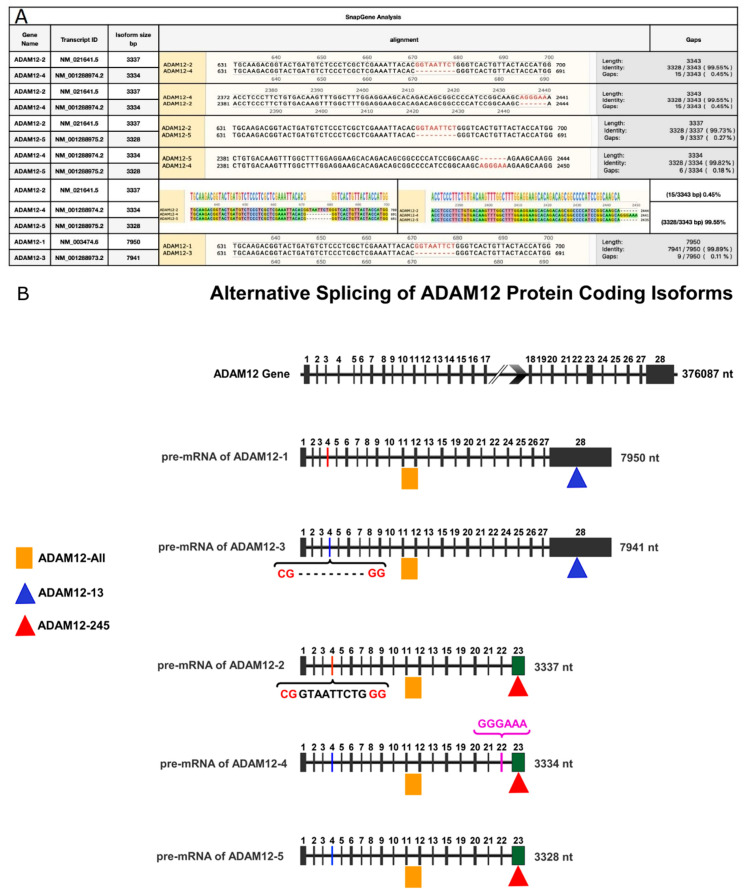
**Sequence similarity analyses for *ADAM12***. (**A**): Sequence similarity of the five alternatively spliced isoforms of *ADAM12*. (**B**): Illustration of the exon organization of *ADAM12* used to design primers for the specific amplification of alternatively spliced isoforms. The *ADAM12* gene and isoforms are presented as horizontal lines. Each vertical bar represents an exon separated by intronic regions. The image shows the skipping of exon 14 in all isoforms and the pretermination of isoforms 2, 4 and 5.
