# Supplementary material for: Alternatively Spliced Isoforms of MUC4 and ADAM12 as Biomarkers for Colorectal Cancer Metastasis
Source: J Pers Med. 2023 Jan 10;13(1):135. doi: 10.3390/jpm13010135 (PMC9861497; doi:10.3390/jpm13010135)
Supplement: Supplementary file 1 [file jpm-13-00135-s001.zip › ADAM12-All-245-13 Sanger Confidence Score.pdf]

Table S1A: Alignment of ADAM12-All Primers using the sample as CCD841, SW480, and SW620 cell lines. Sanger sequencing results were aligned using SnapGene software and NCBI Blast alignment. The confidence scores 99 and 100%.

Gene Name	Cell Line	Amplicon size bp	alignment	Gaps
ADAM12-All Forward	CCD841	198bp	ADAM12-All - CCD841 Amplicon Forward / ADAM12-All Isoforms<br>10 20 30 40 50 60 70 80 90<br>1 CCATTTCCCATGGTGTTCAGCAGTTGCAGCAGGAAGGACTTGGAGACCAGCCTGGAGAAAGGAATGGGGGTGTGCCTGTTTAACCTGCCG 90<br>1469 CCATTTCCCATGGTGTTCAGCAGTTGCAGCAGGAAGGACTTGGAGACCAGCCTGGAGAAAGGAATGGGGGTGTGCCTGTTTAACCTGCCG 1558<br>1470 1480 1490 1500 1510 1520 1530 1540 1550<br>ADAM12-All - CCD841 Amplicon Forward / ADAM12-All Isoforms<br>100 110 120 130 140<br>91 GAAGTCAGGGAGTCTTTCGGGGGCCAGAAGTGTGGGAACAGATTTGTGGA 140<br>1559 GAAGTCAGGGAGTCTTTCGGGGGCCAGAAGTGTGGGAACAGATTTGTGGA 1608<br>1560 1570 1580 1590 1600	Matrix: DNAFULL<br>Gap open penalty: 10.0<br>Gap extend penalty: 1.0<br>Length: 140<br>Identity: 140 / 140 (100.00%)<br>Gaps: 0 / 140 (0.00%)
ADAM12-All Reverse	CCD841	198bp	ADAM12-All - CCD841 Amplicon Reverse / ADAM12-All Isoforms<br>10 20 30 40 50 60 70 80 90<br>1 GTAGCTGTCAAATGGCGGTTGAGAAAGGAGGCTGCATCATGAACGCTTCCACCGGGTACCCATTTCCCATGGTGTTCAGCAGTTGCAGCA 90<br>1432 GTAGCTGTCAAATGGCGGTTGAGAAAGGAGGCTGCATCATGAACGCTTCCACCGGGTACCCATTTCCCATGGTGTTCAGCAGTTGCAGCA 1521<br>1440 1450 1460 1470 1480 1490 1500 1510 1520<br>ADAM12-All - CCD841 Amplicon Reverse / ADAM12-All Isoforms<br>100 110 120 130 140 150<br>91 GGAAGGACTTGGAGACCAGCCTGGAGAAAGGAATGGGGGTGTGCCTG-TTAACCTGCCGGAAGTCAGG 157<br>1522 GGAAGGACTTGGAGACCAGCCTGGAGAAAGGAATGGGGGTGTGCCTGTTTAACCTGCCGGAAGTCAGG 1589<br>1530 1540 1550 1560 1570 1580	Matrix: DNAFULL<br>Gap open penalty: 10.0<br>Gap extend penalty: 1.0<br>Length: 158<br>Identity: 157 / 158 (99.37%)<br>Gaps: 1 / 158 (0.63%)
ADAM12-All Forward	SW480	198bp	ADAM12-All - SW480 Amplicon Forward / ADAM12-All Isoforms<br>10 20 30 40 50 60 70 80<br>1 ACGCTT-CACCGGGTA-CCATTTCCCATGGTGTTCAGCAGTTGCAGCAGGAAGGACTTGGAGACCAGCCTGGAGAAAGGAATGGGGGTGT 88<br>1465 ACGCTTCCACCGGGTACCCATTTCCCATGGTGTTCAGCAGTTGCAGCAGGAAGGACTTGGAGACCAGCCTGGAGAAAGGAATGGGGGTGT 1554<br>1470 1480 1490 1500 1510 1520 1530 1540 1550<br>ADAM12-All - SW480 Amplicon Forward / ADAM12-All Isoforms<br>90 100 110 120 130 140 150<br>89 GCCTGTTTAACCTGCCGGAAGTCAGGGAGTCTTTCGGGGGCCAGAAGTGTGGGAACAGATTTGTGGA 155<br>1555 GCCTGTTTAACCTGCCGGAAGTCAGGGAGTCTTTCGGGGGCCAGAAGTGTGGGAACAGATTTGTGGA 1621<br>1560 1570 1580 1590 1600 1610 1620	Matrix: DNAFULL<br>Gap open penalty: 10.0<br>Gap extend penalty: 1.0<br>Length: 157<br>Identity: 155 / 157 (98.73%)<br>Gaps: 2 / 157 (1.27%)
ADAM12-All Reverse	SW480	198bp	ADAM12-All - Sw480 Amplicon Reverse / ADAM12-All Isoforms<br>10 20 30 40 50 60 70 80 90<br>1 TGTAGCTGTCAAATGGCGGTTGAGAAAGGAGGCTGCATCATGAACGCTTCCACCGGGTACCCATTTCCCATGGTGTTCAGCAGTTGCAGC 90<br>1431 TGTAGCTGTCAAATGGCGGTTGAGAAAGGAGGCTGCATCATGAACGCTTCCACCGGGTACCCATTTCCCATGGTGTTCAGCAGTTGCAGC 1520<br>1440 1450 1460 1470 1480 1490 1500 1510 1520<br>ADAM12-All - Sw480 Amplicon Reverse / ADAM12-All Isoforms<br>100 110 120 130 140 150 160<br>91 AGGAAGGACTTGGAGACCAGCCTGGAGAAAGGAATGGGGGTGTGCCTGTTTAACCTGCCGGAAGTCAGGGAG 162<br>1521 AGGAAGGACTTGGAGACCAGCCTGGAGAAAGGAATGGGGGTGTGCCTGTTTAACCTGCCGGAAGTCAGGGAG 1592<br>1530 1540 1550 1560 1570 1580 1590	Matrix: DNAFULL<br>Gap open penalty: 10.0<br>Gap extend penalty: 1.0<br>Length: 162<br>Identity: 162 / 162 (100.00%)<br>Gaps: 0 / 162 (0.00%)
ADAM12-All Forward	SW620	198bp	ADAM12-All - SW620 Amplicon Forward / ADAM12-All Isoforms<br>10 20 30 40 50 60 70 80 90<br>1 CACCGGGTACCCATTTCCCATGGTGTTCAGCAGTTGCAGCAGGAAGGACTTGGAGACCAGCCTGGAGAAAGGAATGGGGGTGTGCCTGTT 90<br>1481 CACCGGGTACCCATTTCCCATGGTGTTCAGCAGTTGCAGCAGGAAGGACTTGGAGACCAGCCTGGAGAAAGGAATGGGGGTGTGCCTGTT 1570<br>1490 1500 1510 1520 1530 1540 1550 1560 1570<br>ADAM12-All - SW620 Amplicon Forward / ADAM12-All Isoforms<br>100 110 120 130 140<br>91 TAACCTGCCGGAAGTCAGGGAGTCTTTCGGGGGCCAGAAGTGTGGGAACAGATTTGTGG 149<br>1571 TAACCTGCCGGAAGTCAGGGAGTCTTTCGGGGGCCAGAAGTGTGGGAACAGATTTGTGG 1629<br>1580 1590 1600 1610 1620	Matrix: DNAFULL<br>Gap open penalty: 10.0<br>Gap extend penalty: 1.0<br>Length: 149<br>Identity: 149 / 149 (100.00%)<br>Gaps: 0 / 149 (0.00%)
ADAM12-All Reverse	SW620	198bp	ADAM12-All - SW620 Amplicon Reverse / ADAM12-All Isoforms<br>10 20 30 40 50 60 70 80 90<br>1 GTAGCTGTCAAATGGCGGTTGAGAAAGGAGGCTGCATCATGAACGCTTCCACCGGGTACCCATTTCCCATGGTGTTCAGCAGTTGCAGCA 90<br>1432 GTAGCTGTCAAATGGCGGTTGAGAAAGGAGGCTGCATCATGAACGCTTCCACCGGGTACCCATTTCCCATGGTGTTCAGCAGTTGCAGCA 1521<br>1440 1450 1460 1470 1480 1490 1500 1510 1520<br>ADAM12-All - SW620 Amplicon Reverse / ADAM12-All Isoforms<br>100 110 120 130 140 150<br>91 GGAAGGACTTGGAGACCAGCCTGGAGAAAGGAATGGGGGTGTGCCTG-TTAACCTGCCGGAAGTCA 155<br>1522 GGAAGGACTTGGAGACCAGCCTGGAGAAAGGAATGGGGGTGTGCCTGTTTAACCTGCCGGAAGTCA 1587<br>1530 1540 1550 1560 1570 1580	Matrix: DNAFULL<br>Gap open penalty: 10.0<br>Gap extend penalty: 1.0<br>Length: 156<br>Identity: 155 / 156 (99.36%)<br>Gaps: 1 / 156 (0.64%)

Table S1B: Alignment of ADAM12-245 Primers using the sample as CCD841, SW480, and SW620 cell lines. Sanger sequencing results were aligned using SnapGene software and NCBI Blast alignment. The confidence scores 99 and 100%.

Gene Name	Cell Line	Amplicon size bp	alignment	Gaps
**ADAM12-245 Forward**	**CCD841**	**205bp**	ADAM12-245 - CCD841 Amplicon Forward<br>ADAM12-245 Isoforms<br>10 20 30 40 50 60 70 80 90 100<br>1 AGGAGGAAGAGGACTCAAAAGTCTGGCCTTTCACTGAGCCCCCACAGCAGTGGGGGAGAAGCAAGGGTTGGGCCCAGTGTCCCCTTTCCCCAGTGACACC 100<br>2847 AGGAGGAAGAGGACTCAAAAGTCTGGCCTTTCACTGAGCCTCCACAGCAGTGGGGGAGAAGCAAGGGTTGGGCCCAGTGTCCCCTTTCCCCAGTGACACC 2946<br>2850 2860 2870 2880 2890 2900 2910 2920 2930 2940<br>ADAM12-245 - CCD841 Amplicon Forward<br>ADAM12-245 Isoforms<br>110 120 130 140 150<br>101 TCAGCCTTGGCAGCCCTGATGACTGGTCTCTGGCTGCAACTTAATGCTCTGA 152<br>2947 TCAGCCTTGGCAGCCCTGATGACTGGTCTCTGGCTGCAACTTAATGCTCTGA 2998<br>2950 2960 2970 2980 2990	Matrix: DNAFULL<br>Gap open penalty: 10.0<br>Gap extend penalty: 1.0<br>Length: 152<br>Identity: 151 / 152 (99.34%)<br>Gaps: 0 / 152 (0.00%)
**ADAM12-245 Reverse**	**CCD841**	**205bp**	ADAM12-245 - CCD841 Amplicon Reverse<br>ADAM12-245 Isoforms<br>10 20 30 40 50 60 70 80 90 100<br>1 CTGCTCCTGAGAGAGTAGCAGGTTACCACTCTGGCAGGCCCCAGCCCTGCAGCAAGGAGGAAGAGGACTCAAAAGTCTGGCCTTTCACTGAGCCTCCACA 100<br>2793 CTGCTCCTGAGAGAGTAGCAGGTTACCACTCTGGCAGGCCCCAGCCCTGCAGCAAGGAGGAAGAGGACTCAAAAGTCTGGCCTTTCACTGAGCCTCCACA 2892<br>2800 2810 2820 2830 2840 2850 2860 2870 2880 2890<br>ADAM12-245 - CCD841 Amplicon Reverse<br>ADAM12-245 Isoforms<br>110 120 130 140 150 160<br>101 GCAGTGGGGGAGAAGCAAGGGTTGGGCCCAGTGTCCCCTTTCCCCAGTGACACCTCAGCCT 161<br>2893 GCAGTGGGGGAGAAGCAAGGGTTGGGCCCAGTGTCCCCTTTCCCCAGTGACACCTCAGCCT 2953<br>2900 2910 2920 2930 2940 2950	Matrix: DNAFULL<br>Gap open penalty: 10.0<br>Gap extend penalty: 1.0<br>Length: 161<br>Identity: 161 / 161 (100.00%)<br>Gaps: 0 / 161 (0.00%)
**ADAM12-245 Forward**	**SW480**	**205bp**	ADAM12-245 - SW480 Amplicon Forward<br>ADAM12-245 Isoforms<br>10 20 30 40 50 60 70 80 90 100<br>1 CCAGCCCTGCAGCAAGGAGGAAGAGGACTCAAAAGTCTGGCCTTTCACTGAGCCTCCACAGCAGTGGGGGAGAAGCAAGGGTTGGGCCCAGTGTCCCCTT 100<br>2833 CCAGCCCTGCAGCAAGGAGGAAGAGGACTCAAAAGTCTGGCCTTTCACTGAGCCTCCACAGCAGTGGGGGAGAAGCAAGGGTTGGGCCCAGTGTCCCCTT 2932<br>2840 2850 2860 2870 2880 2890 2900 2910 2920 2930<br>ADAM12-245 - SW480 Amplicon Forward<br>ADAM12-245 Isoforms<br>110 120 130 140 150 160<br>101 TCCCCAGTGACACCTCAGCCTTGGCAGCCCTGATGACTGGTCTCTGGCTGCAACTTAATGCTCTGA 166<br>2933 TCCCCAGTGACACCTCAGCCTTGGCAGCCCTGATGACTGGTCTCTGGCTGCAACTTAATGCTCTGA 2998<br>2940 2950 2960 2970 2980 2990	Matrix: DNAFULL<br>Gap open penalty: 10.0<br>Gap extend penalty: 1.0<br>Length: 166<br>Identity: 166 / 166 (100.00%)<br>Gaps: 0 / 166 (0.00%)
**ADAM12-245 Reverse**	**SW480**	**205bp**	ADAM12-245 - SW480 Amplicon Reverse<br>ADAM12-245 Isoforms<br>10 20 30 40 50 60 70 80 90 100<br>1 CTGCTCCTGAGAGAGTAGCAGGTTACCACTCTGGCAGGCCCCAGCCCTGCAGCAAGGAGGAAGAGGACTCAAAAGTCTGGCCTTTCACTGAGCCTCCACA 100<br>2793 CTGCTCCTGAGAGAGTAGCAGGTTACCACTCTGGCAGGCCCCAGCCCTGCAGCAAGGAGGAAGAGGACTCAAAAGTCTGGCCTTTCACTGAGCCTCCACA 2892<br>2800 2810 2820 2830 2840 2850 2860 2870 2880 2890<br>ADAM12-245 - SW480 Amplicon Reverse<br>ADAM12-245 Isoforms<br>110 120 130 140 150 160<br>101 AAGAGGACTCAAAAGTCTGGCCTTTCACTGAGCCTCCACAGCAGTGGGGGAGAAGCAAGG 160<br>2893 AAGAGGACTCAAAAGTCTGGCCTTTCACTGAGCCTCCACAGCAGTGGGGGAGAAGCAAGG 2952<br>2900 2910 2920 2930 2940 2950	Matrix: DNAFULL<br>Gap open penalty: 10.0<br>Gap extend penalty: 1.0<br>Length: 160<br>Identity: 160 / 160 (100.00%)<br>Gaps: 0 / 160 (0.00%)
**ADAM12-245 Forward**	**SW620**	**205bp**	ADAM12-245 - SW620 Amplicon Forward<br>ADAM12-245 Isoforms<br>10 20 30 40 50 60 70 80 90<br>1 CCAGCCCTGCAGC-AGGAGGAAGAGGACTCAAAAGTCTGGCCTTTCACTGAGCCTCCACAGCAGTGGGGGAGAAGCAAGGGTTGGGCCCAGTGTCCCCTT 99<br>2833 CCAGCCCTGCAGCAAGGAGGAAGAGGACTCAAAAGTCTGGCCTTTCACTGAGCCTCCACAGCAGTGGGGGAGAAGCAAGGGTTGGGCCCAGTGTCCCCTT 2932<br>2840 2850 2860 2870 2880 2890 2900 2910 2920 2930<br>ADAM12-245 - SW620 Amplicon Forward<br>ADAM12-245 Isoforms<br>100 110 120 130 140 150 160<br>100 TCCCCAGTGACACCTCAGCCTTGGCAGCCCTGATGACTGGTCTCTGGCTGCAACTTAATGCTCTGA 165<br>2933 TCCCCAGTGACACCTCAGCCTTGGCAGCCCTGATGACTGGTCTCTGGCTGCAACTTAATGCTCTGA 2998<br>2940 2950 2960 2970 2980 2990	Matrix: DNAFULL<br>Gap open penalty: 10.0<br>Gap extend penalty: 1.0<br>Length: 166<br>Identity: 165 / 166 (99.40%)<br>Gaps: 1 / 166 (0.60%)
**ADAM12-245 Reverse**	**SW620**	**205bp**	ADAM12-245 - SW620 Amplicon Reverse<br>ADAM12-245 Isoforms<br>10 20 30 40 50 60 70 80 90 100<br>1 CTGCTCCTGAGAGAGTAGCAGGTTACCACTCTGGCAGGCCCCAGCCCTGCAGCAAGGAGGAAGAGGACTCAAAAGTCTGGCCTTTCACTGAGCCTCCACA 100<br>2793 CTGCTCCTGAGAGAGTAGCAGGTTACCACTCTGGCAGGCCCCAGCCCTGCAGCAAGGAGGAAGAGGACTCAAAAGTCTGGCCTTTCACTGAGCCTCCACA 2892<br>2800 2810 2820 2830 2840 2850 2860 2870 2880 2890<br>ADAM12-245 - SW620 Amplicon Reverse<br>ADAM12-245 Isoforms<br>110 120 130 140 150 160<br>101 GCAGTGGGGGAGAAGCAAGGGTTGGGCCCAGTGTCCCCTTTCCCCAGTGACACCTCAGCC 160<br>2893 GCAGTGGGGGAGAAGCAAGGGTTGGGCCCAGTGTCCCCTTTCCCCAGTGACACCTCAGCC 2952<br>2900 2910 2920 2930 2940 2950	Matrix: DNAFULL<br>Gap open penalty: 10.0<br>Gap extend penalty: 1.0<br>Length: 160<br>Identity: 160 / 160 (100.00%)<br>Gaps: 0 / 160 (0.00%)

Table S1C: Alignment of ADAM12-245 Primers using the sample as CCD841, SW480, and SW620 cell lines. Sanger sequencing results were aligned using SnapGene software and NCBI Blast alignment. The confidence scores 99 and 100%.

Gene Name	Cell Line	Amplicon size bp	alignment	Gaps
**ADAM12-13 Forward**	**CCD841**	**197bp**	ADAM12-13 - CCD841 Amplicon Forward<br>ADAM12-13 Isoforms<br>10 20 30 40 50 60 70 80 90 100<br>1 AATGTAAAGCCATGCTGGAAAATAATACTGCTGAGATACATACAGAATTACTGTAACTGATTACACTTGGTAATTGTACTAAAGCCAAACATATATATAC 100<br>4855 AATGTAAAGCCATGCTGGAAAATAATACTGCTGAGATACATACAGAATTACTGTAACTGATTACACTTGGTAATTGTACTAAAGCCAAACATATATATAC 4954<br>4860 4870 4880 4890 4900 4910 4920 4930 4940 4950<br>ADAM12-13 - CCD841 Amplicon Forward<br>ADAM12-13 Isoforms<br>110 120 130 140<br>101 TATTAAAAAGGTTTACAGAATTTTATGGTGCATTACGTGGGCATTG 146<br>4955 TATTAAAAAGGTTTACAGAATTTTATGGTGCATTACGTGGGCATTG 5000<br>4960 4970 4980 4990 5000	Matrix: DNAFULL<br>Gap open penalty: 10.0<br>Gap extend penalty: 1.0<br>Length: 146<br>Identity: 146 / 146 (100.00%)<br>Gaps: 0 / 146 (0.00%)
**ADAM12-13 Reverse**	**CCD841**	**197bp**	ADAM12-13 - CCD841 Amplicon Reverse<br>ADAM12-13 Isoforms<br>10 20 30 40 50 60 70 80 90 100<br>1 TCCAACTCGTATAGCATGCATCTGTTTATTCTATAGTTATTAAGTTCTTTAAAATGTAAAGCCATGCTGGAAAATAATACTGCTGAGATACATACAGAAT 100<br>4803 TCCAACTCGTATAGCATGCATCTGTTTATTCTATAGTTATTAAGTTCTTTAAAATGTAAAGCCATGCTGGAAAATAATACTGCTGAGATACATACAGAAT 4902<br>4810 4820 4830 4840 4850 4860 4870 4880 4890 4900<br>ADAM12-13 - CCD841 Amplicon Reverse<br>ADAM12-13 Isoforms<br>110 120 130 140 150 160<br>101 TACTGTAACTGATTACACTTGGTAATTGTACTAAAGCCAAACATATATATACTATAAAAAAGGTT 165<br>4903 TACTGTAACTGATTACACTTGGTAATTGTACTAAAGCCAAACATATATATACTATTAAAAAGGTT 4967<br>4910 4920 4930 4940 4950 4960	Matrix: DNAFULL<br>Gap open penalty: 10.0<br>Gap extend penalty: 1.0<br>Length: 165<br>Identity: 164 / 165 (99.39%)<br>Gaps: 0 / 165 (0.00%)
**ADAM12-13 Forward**	**SW480**	**197bp**	ADAM12-13 - SW480 Amplicon Forward<br>ADAM12-13 Isoforms<br>10 20 30 40 50 60 70 80 90 100<br>1 AAATGTAAAGCCATGCTGGAAAATAATACTGCTGAGATACATACAGAATTACTGTAACTGATTACACTTGGTAATTGTACTAAAGCCAAACATATATATA 100<br>4854 AAATGTAAAGCCATGCTGGAAAATAATACTGCTGAGATACATACAGAATTACTGTAACTGATTACACTTGGTAATTGTACTAAAGCCAAACATATATATA 4953<br>4860 4870 4880 4890 4900 4910 4920 4930 4940 4950<br>ADAM12-13 - SW480 Amplicon Forward<br>ADAM12-13 Isoforms<br>110 120 130 140<br>101 CTATTAAAAAGGTTTACAGAATTTTATGGTGCATTACGTGGGCATTG 147<br>4954 CTATTAAAAAGGTTTACAGAATTTTATGGTGCATTACGTGGGCATTG 5000<br>4960 4970 4980 4990 5000	Matrix: DNAFULL<br>Gap open penalty: 10.0<br>Gap extend penalty: 1.0<br>Length: 147<br>Identity: 147 / 147 (100.00%)<br>Gaps: 0 / 147 (0.00%)
**ADAM12-13 Reverse**	**SW480**	**197bp**	ADAM12-13 - SW480 Amplicon Reverse<br>ADAM12-13 Isoforms<br>10 20 30 40 50 60 70 80 90 100<br>1 TCCAACTCGTATAGCATGCATCTGTTTATTCTATAGTTATTAAGTTCTTTAAAATGTAAAGCCATGCTGGAAAATAATACTGCTGAGATACATACAGAAT 100<br>4803 TCCAACTCGTATAGCATGCATCTGTTTATTCTATAGTTATTAAGTTCTTTAAAATGTAAAGCCATGCTGGAAAATAATACTGCTGAGATACATACAGAAT 4902<br>4810 4820 4830 4840 4850 4860 4870 4880 4890 4900<br>ADAM12-13 - SW480 Amplicon Reverse<br>ADAM12-13 Isoforms<br>110 120 130 140 150<br>101 TACTGTAACTGATTACACTTGGTAATTGTACTAAAGCCAAACATATATATACT 153<br>4903 TACTGTAACTGATTACACTTGGTAATTGTACTAAAGCCAAACATATATATACT 4955<br>4910 4920 4930 4940 4950	Matrix: DNAFULL<br>Gap open penalty: 10.0<br>Gap extend penalty: 1.0<br>Length: 153<br>Identity: 153 / 153 (100.00%)<br>Gaps: 0 / 153 (0.00%)
**ADAM12-13 Forward**	**SW620**	**197bp**	ADAM12-13 - SW620 Amplicon Forward<br>ADAM12-13 Isoforms<br>10 20 30 40 50 60 70 80 90 100<br>1 AATGTAAAGCCATGCTGGAAAATAATACTGCTGAGATACATACAGAATTACTGTAACTGATTACACTTGGTAATTGTACTAAAGCCAAACATATATATAC 100<br>4855 AATGTAAAGCCATGCTGGAAAATAATACTGCTGAGATACATACAGAATTACTGTAACTGATTACACTTGGTAATTGTACTAAAGCCAAACATATATATAC 4954<br>4860 4870 4880 4890 4900 4910 4920 4930 4940 4950<br>ADAM12-13 - SW620 Amplicon Forward<br>ADAM12-13 Isoforms<br>110 120 130 140<br>101 TATTAAAAAGGTTTACAGAATTTTATGGTGCATTACGTGGGCATT 145<br>4955 TATTAAAAAGGTTTACAGAATTTTATGGTGCATTACGTGGGCATT 4999<br>4960 4970 4980 4990	Matrix: DNAFULL<br>Gap open penalty: 10.0<br>Gap extend penalty: 1.0<br>Length: 145<br>Identity: 145 / 145 (100.00%)<br>Gaps: 0 / 145 (0.00%)
**ADAM12-13 Reverse**	**SW620**	**197bp**	ADAM12-13 - SW620 Amplicon Reverse<br>ADAM12-13 Isoforms<br>10 20 30 40 50 60 70 80 90 100<br>1 TCCAACTCGTATAGCATGCATCTGTTTATTCTATAGTTATTAAGTTCTTTAAAATGTAAAGCCATGCTGGAAAATAATACTGCTGAGATACATACAGAAT 100<br>4803 TCCAACTCGTATAGCATGCATCTGTTTATTCTATAGTTATTAAGTTCTTTAAAATGTAAAGCCATGCTGGAAAATAATACTGCTGAGATACATACAGAAT 4902<br>4810 4820 4830 4840 4850 4860 4870 4880 4890 4900<br>ADAM12-13 - SW620 Amplicon Reverse<br>ADAM12-13 Isoforms<br>110 120 130 140 150<br>101 TACTGTAACTGATTACACTTGGTAATTGTACTAAAGCCAAACATATATATACTAT 155<br>4903 TACTGTAACTGATTACACTTGGTAATTGTACTAAAGCCAAACATATATATACTAT 4957<br>4910 4920 4930 4940 4950	Matrix: DNAFULL<br>Gap open penalty: 10.0<br>Gap extend penalty: 1.0<br>Length: 155<br>Identity: 155 / 155 (100.00%)<br>Gaps: 0 / 155 (0.00%)
