# Supplementary material for: Alternatively Spliced Isoforms of MUC4 and ADAM12 as Biomarkers for Colorectal Cancer Metastasis
Source: J Pers Med. 2023 Jan 10;13(1):135. doi: 10.3390/jpm13010135 (PMC9861497; doi:10.3390/jpm13010135)
Supplement: Supplementary file 1 [file jpm-13-00135-s001.zip › MUC4-6 & MUC4-1 copy.pdf]

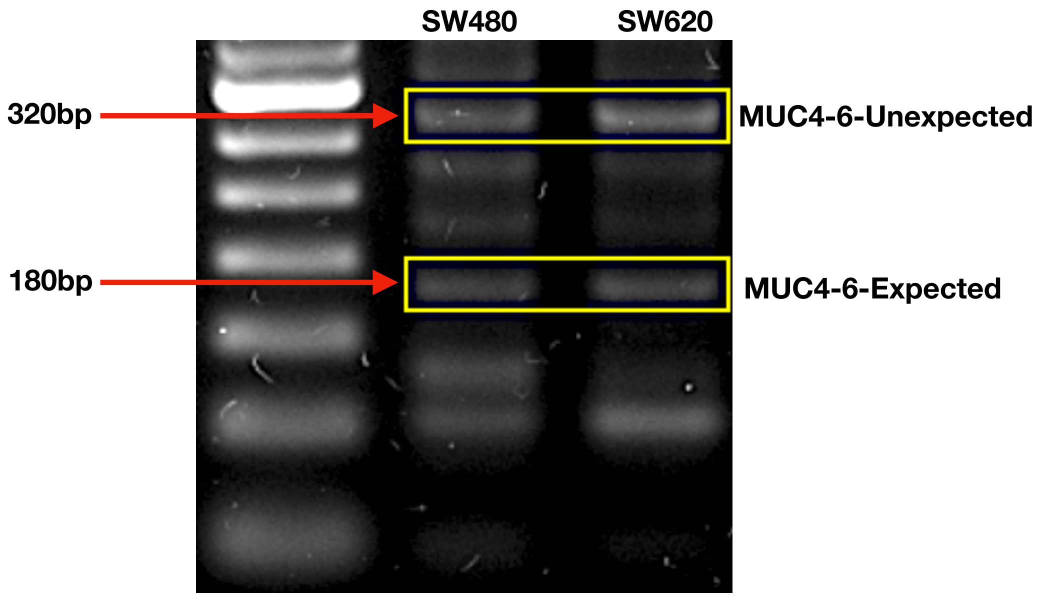


**Supplementary Figure S3A: The primers that were designed to detect MUC4-6 showed multiple bands. The yellow box showed the excised bands. The templates were the cell lines SW480 and SW620.**

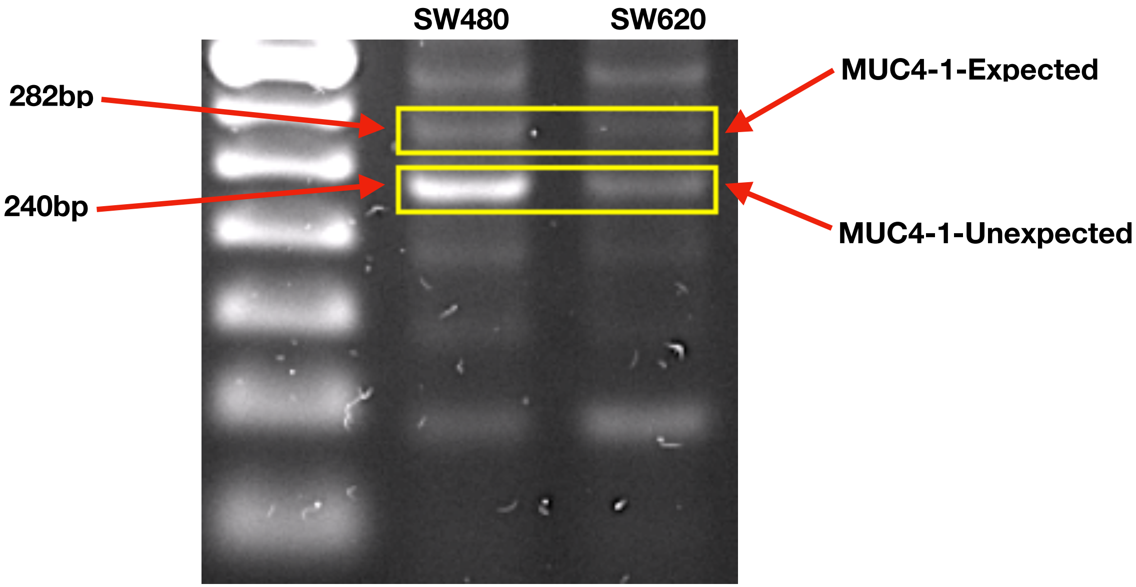


**Supplementary Figure S3A: The primers that were designed to detect MUC4-1 showed multiple bands. The yellow box showed the excised bands. The templates were the cell lines SW480 and SW620.**
