# Supplementary material for: Alternatively Spliced Isoforms of MUC4 and ADAM12 as Biomarkers for Colorectal Cancer Metastasis
Source: J Pers Med. 2023 Jan 10;13(1):135. doi: 10.3390/jpm13010135 (PMC9861497; doi:10.3390/jpm13010135)
Supplement: Supplementary file 1 [file jpm-13-00135-s001.zip › MUC4-6 & MUC4-1.pdf]

Table S3A: Alignment of MUC4-6 primers using the sample as CCD841, SW480, and SW620 cell lines. Sanger sequencing results were aligned using SnapGene software and NCBI Blast alignment. The confidence scores 80 and 100%.

Gene Name	Cell Line	Amplicon size bp	alignment	Gaps
MUC4-6 Forward	CCD841	180bp	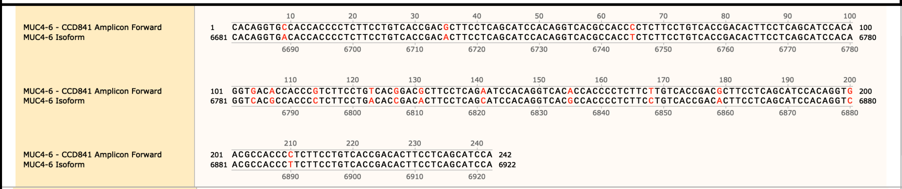	Matrix: DNAFULL<br>Gap open penalty: 10.0<br>Gap extend penalty: 1.0<br>Length: 242<br>Identity: 227 / 242 (93.80%)<br>Gaps: 0 / 242 (0.00%)
MUC4-6 Forward	CCD841	320bp	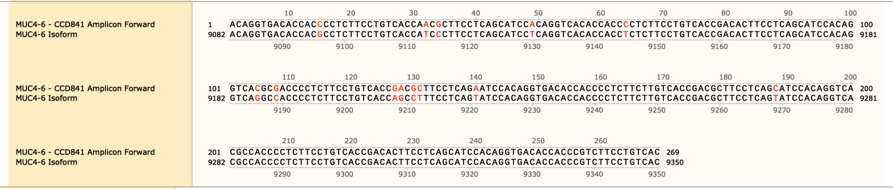	Matrix: DNAFULL<br>Gap open penalty: 10.0<br>Gap extend penalty: 1.0<br>Length: 269<br>Identity: 256 / 269 (95.17 %)<br>Gaps: 0 / 269 (0.00%)
MUC4-6 Forward	SW480	180bp	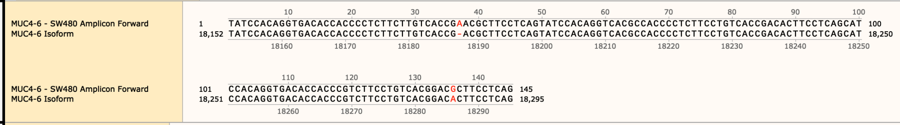	Matrix: DNAFULL<br>Gap open penalty: 10.0<br>Gap extend penalty: 1.0<br>Length: 145<br>Identity: 143 / 145 (98.62%)<br>Gaps: 1 / 145 (0.69%)
MUC4-6 Forward	SW480	320bp	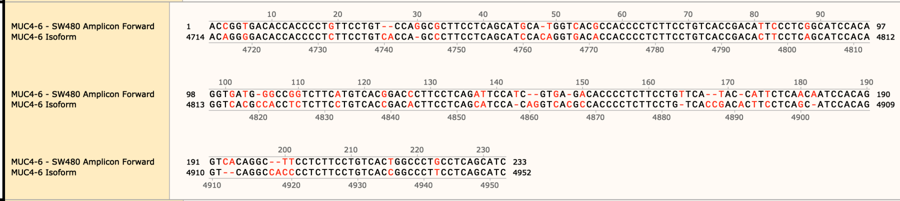	Matrix: DNAFULL<br>Gap open penalty: 10.0<br>Gap extend penalty: 1.0<br>Length: 245<br>Identity: 195 / 245 (79.59%)<br>Gaps: 18 / 245 (7.35%)
MUC4-6 Forward	SW620	180bp	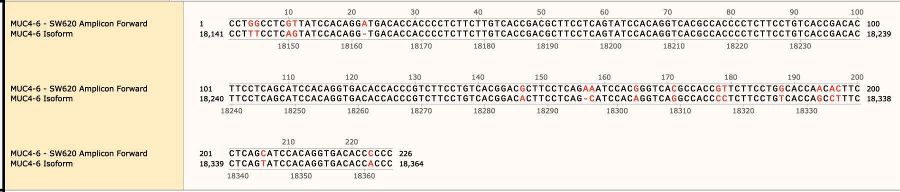	Matrix: DNAFULL<br>Gap open penalty: 10.0<br>Gap extend penalty: 1.0<br>Length: 226<br>Identity: 208 / 226 (92.04%)<br>Gaps: 2 / 226 (0.88%)
MUC4-6 Reverse	SW620	180bp	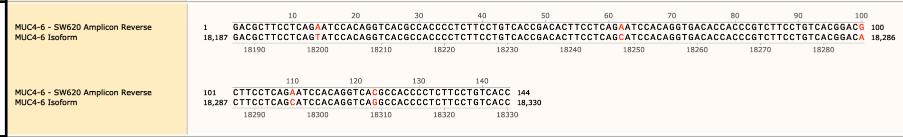	Matrix: DNAFULL<br>Gap open penalty: 10.0<br>Gap extend penalty: 1.0<br>Length: 144<br>Identity: 139 / 144 (96.53%)<br>Gaps: 0 / 144 (0.00%)
MUC4-6 Forward	SW620	320bp	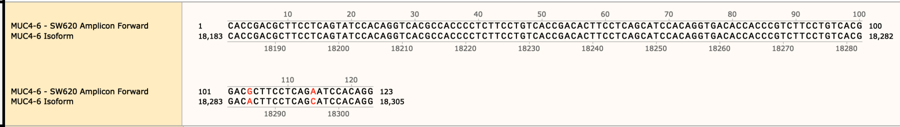	Matrix: DNAFULL<br>Gap open penalty: 10.0<br>Gap extend penalty: 1.0<br>Length: 123<br>Identity: 121 / 123 (98.37 %)<br>Gaps: 0 / 123 (0.00%)
MUC4-6 Reverse	SW620	320bp	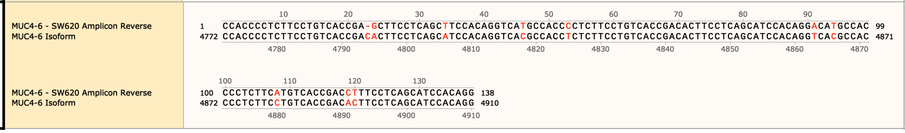	Matrix: DNAFULL<br>Gap open penalty: 10.0<br>Gap extend penalty: 1.0<br>Length: 139<br>Identity: 129 / 139 (92.81 %)<br>Gaps: 1 / 139 (0.72 %)

Table S3B: Alignment of MUC4-1 primers using the sample as CCD841, SW480, and SW620 cell lines. Sanger sequencing results were aligned using SnapGene software and NCBI Blast alignment. The confidence scores 91 and 100%.

Gene Name	Cell Line	Amplicon size bp	alignment	Gaps
MUC4-1 Forward	CCD841	282bp	MUC4-1 - CCD841 Amplicon Forward<br>MUC4-1 Isoform<br>10 20 30 40 50 60 70 80 90 100<br>1 ACACTTACTCAGCATCCACAGGACACGCCACCCCTCTTCCTGTCACCGACACTTCCTCAGCGTCCACAGGTGACGCCACCCCTCTTCCTGTCACCGACAC 100<br>3390 ACACTTCCTCAGCATCCACAGGTCACGCCACCTCTCTTCCTGTCACCGACACTTCCTCAGTATCCACAGGTCACACCACCCCTCTTCCTGTCACCGACAC 3489<br>3390 3400 3410 3420 3430 3440 3450 3460 3470 3480<br>MUC4-1 - CCD841 Amplicon Forward<br>MUC4-1 Isoform<br>110 120 130 140 150 160<br>101 TTCCTCAGCATCCACAGGTCACGCCACCCCTCTTCCTGTCACCGACACTTCCTCAGTATCCACAGG 166<br>3490 TTCCTCAGCATCCACAGGTCACGCCACCTCTCTTCCTGTCACCGACACTTCCTCAGTATCCACAGG 3555<br>3490 3500 3510 3520 3530 3540 3550	Matrix: DNAFULL<br>Gap open penalty: 10.0<br>Gap extend penalty: 1.0<br>Length: 166<br>Identity: 158 / 166 (95.18 %)<br>Gaps: 0 / 166 (0.00%)
MUC4-1 Forward	CCD841	240bp	MUC4-1 - CCD841 Amplicon Forward<br>MUC4-1 Isoform<br>10 20 30 40 50 60 70 80 90<br>1 TCTTTCTGTCA-CGACACTTCCTCAGTATCCACAGGTCACGCCACCTCTCTTCCTGTCACCGACGCTTCCTCAGTGTCCACAGGTCACGCCACCCCTCTT 99<br>5968 TCTTCCTGTCACCGACGCTTCCTCAGTACCCACAGGTCACGCCACCTCTCTTCCTGTCACCGACGCTTCCTCAGTGTCCACAGGTCACGCCACCCCTCTT 6067<br>5970 5980 5990 6000 6010 6020 6030 6040 6050 6060<br>MUC4-1 - CCD841 Amplicon Forward<br>MUC4-1 Isoform<br>100 110 120 130 140 150 160 170 180 190<br>100 CCTGTCACCGACACTTCCTCAGTGTCCACAGGTCATGCCACCCCTCTTCCGGTCACCGACACTTCCTCAGTATCTACAGGACATGCCACCCCTCTTC 196<br>6068 CCTGTCACCGACGCTTCCTCAGTGTCCACAGGTCATGCCACCCCTCTTCCGGTCACCGACACTTCCTCAGTATCTACAGGACAGGCCACCCCTCTTC 6164<br>6070 6080 6090 6100 6110 6120 6130 6140 6150 6160	Matrix: DNAFULL<br>Gap open penalty: 10.0<br>Gap extend penalty: 1.0<br>Length: 197<br>Identity: 191 / 197 (96.95%)<br>Gaps: 1 / 197 (0.51 %)
MUC4-1 Reverse	CCD841	240bp	MUC4-1 - CCD841 Amplicon Reverse<br>MUC4-1 Isoform<br>10 20 30 40 50 60 70 80 90 100<br>1 TCCTCAGTGTCCACAGGGGACGCCACCCCTCTTCCTGTTACCGACATTTCCTCAGCGTCCACAGGTGACGCCACCCCTCTTCCTGTCACCGACACTTCCT 100<br>6563 TCCTCAGTATCCACAGGTCACGCCACCTCTCTTCCTGTCACCGACACTTCCTCAGCATCCACAGGTCACGCCACCTCTCTTCCTGTCACCGACACTTCCT 6662<br>6570 6580 6590 6600 6610 6620 6630 6640 6650 6660<br>MUC4-1 - CCD841 Amplicon Reverse<br>MUC4-1 Isoform<br>110 120 130 140 150 160 170 180<br>101 CAGCATCCACAGGTGATGCCACCCCTCTTCCTGTCACCGACACTTCCTCAGTATCCACAGGACATGCCACCCCTCTTCATGTCACC 186<br>6663 CAGCATCCACAGGTCACGCCACCCCTCTTCCTGTCACCGACACTTCCTCAGCATCCACAGGTCAGGCCACCCCTCTTCCTGTCACC 6748<br>6670 6680 6690 6700 6710 6720 6730 6740	Matrix: DNAFULL<br>Gap open penalty: 10.0<br>Gap extend penalty: 1.0<br>Length: 186<br>Identity: 171 / 186 (91.94%)<br>Gaps: 0 / 186 (0.00%)
MUC4-1 Forward	SW480	240bp	MUC4-1 - SW480 Amplicon Forward<br>MUC4-1 Isoform<br>10 20 30 40 50 60 70 80 90<br>1 TCTTCCTGTCA-CGACACTTCCTCAGTATCCACAGGTCACGCCACCTCTCTTCCTGTCACCGACGCTTCCTCAGTGTCCACAGGTCACGCCACCCCTCTT 99<br>5968 TCTTCCTGTCACCGACGCTTCCTCAGTACCCACAGGTCACGCCACCTCTCTTCCTGTCACCGACGCTTCCTCAGTGTCCACAGGTCACGCCACCCCTCTT 6067<br>5970 5980 5990 6000 6010 6020 6030 6040 6050 6060<br>MUC4-1 - SW480 Amplicon Forward<br>MUC4-1 Isoform<br>100 110 120 130 140 150 160 170 180 190<br>100 CCTGTCACCGACACTTCCTCAGTGTCCACAGGTCATGCCACCCCTCTTCCTGTCACCGACACTTCCTCAGTATCTACAGGACATGCCACCCCTCTTC 196<br>6068 CCTGTCACCGACGCTTCCTCAGTGTCCACAGGTCATGCCACCCCTCTTCCGGTCACCGACACTTCCTCAGTATCTACAGGACAGGCCACCCCTCTTC 6164<br>6070 6080 6090 6100 6110 6120 6130 6140 6150 6160	Matrix: DNAFULL<br>Gap open penalty: 10.0<br>Gap extend penalty: 1.0<br>Length: 197<br>Identity: 191 / 197 (96.95%)<br>Gaps: 1 / 197 (0.51 %)
MUC4-1 Forward	SW480	282bp	MUC4-1 - SW480 Amplicon Forward<br>MUC4-1 Isoform<br>10 20 30 40 50 60 70 80 90 100<br>1 TCCACAGGTGACGCCACCCCTCTTCCTGTCACCGACACTTCCTCAGCATCCACAGGTCATGCCACCCCTCTTCCTGTCACCGACACTTCCTCAGCATCCA 100<br>3356 TCCACAGGTGACACCACCCCTCTTCCTGTCACTGACACTTCCTCAGCATCCACAGGTCACGCCACCTCTCTTCCTGTCACCGACACTTCCTCAGTATCCA 3455<br>3360 3370 3380 3390 3400 3410 3420 3430 3440 3450<br>MUC4-1 - SW480 Amplicon Forward<br>MUC4-1 Isoform<br>110 120 130 140 150 160 170 180 190 200<br>101 CAGGACATGCCACCCCTCTTCATGTCACCGACACTTCCTCAGCATCCACAGGACATGCCACCCCTCTTCATGTCACCGACACTTCCTCAGCATCCACAGG 200<br>3456 CAGGTCACACCACCCCTCTTCCTGTCACCGACACTTCCTCAGCATCCACAGGTCACGCCACCTCTCTTCCTGTCACCGACACTTCCTCAGTATCCACAGG 3555<br>3460 3470 3480 3490 3500 3510 3520 3530 3540 3550<br>MUC4-1 - SW480 Amplicon Forward<br>MUC4-1 Isoform<br>210 220<br>201 ACACACCACCCCTCTTCATGT 221<br>3556 TCACACCACCCCTCTTCATGT 3576<br>3560 3570	Matrix: DNAFULL<br>Gap open penalty: 10.0<br>Gap extend penalty: 1.0<br>Length: 221<br>Identity: 206 / 221 (93.21 %)<br>Gaps: 0 / 221 (0.00%)
MUC4-1 Reverse	SW620	282bp	MUC4-1 - SW620 Amplicon Forward<br>MUC4-1 Isoform<br>10 20 30 40 50 60 70 80 90 100<br>1 TTCCTCAGCATCCACAGGTCACGCCACCCCTCTTCCTGTCACCGACACTTCCTCAGCATCCACAGGTCACGCCACCCCTCTTCCTGTCACCGACACTTCC 100<br>8914 TTCCTCAGTATCCACAGGTGACACCACCCCTCTTCCTGTCACCGACACTTCCTCAGCATCCACAGGTCACGCCACCTCTCTTCCTGTCACCGACACTTCC 9013<br>8920 8930 8940 8950 8960 8970 8980 8990 9000 9010<br>MUC4-1 - SW620 Amplicon Forward<br>MUC4-1 Isoform<br>110 120 130 140 150 160 170 180 190 200<br>101 TCAGCATCCACAGGTCACGCCACCCCTCTTCCTGTCACCGACACTTCCTCAGCATCCACAGGTCATGCCACCCCTCTTCATGTCACCGACACTTCCTCAG 200<br>9014 TCAGCATCCACAGGTCACGCCACCCCTCTTCCTGACACCGACACTTCCTCAGCATCCACAGGTCACGCCACCCTTCTTCCTGTCACCGACACTTCCTCAG 9113<br>9020 9030 9040 9050 9060 9070 9080 9090 9100 9110<br>MUC4-1 - SW620 Amplicon Forward<br>MUC4-1 Isoform<br>210 220 230 240 250 260 270 280 290 300<br>201 CATCCACAGGACATGCCACCCCTCTTCATGTCACCGACACTTCCTCAGCATCCACAGGACATGCCACCCCTCTTCATGTCACCGACACTTCCTCAGCATC 300<br>9114 CATCCATAGGTCACGCCACCTCTCTTCCTGTCACCGACACTTCCTCAATATCCACAGGTCACGCCACCCCTCTTCATGTCACCAGCCCTTCCTCAGCATC 9213<br>9120 9130 9140 9150 9160 9170 9180 9190 9200 9210<br>MUC4-1 - SW620 Amplicon Forward<br>MUC4-1 Isoform<br>310 320 330 340 350 360 370<br>301 CACAGGTCACGCCACCCCTCTTCATGTCACCGACACTTCCTCAGCATCCACAGGACACGCCACCCCTCTTCATGTCACC 379<br>9214 CACCGGTCACGCCACCCCGCTTCCTGTCACCGACACTTCCTCAGCATCCACAGGTCACGCCAACCCTCTTCATGTCACC 9292<br>9220 9230 9240 9250 9260 9270 9280 9290	Matrix: DNAFULL<br>Gap open penalty: 10.0<br>Gap extend penalty: 1.0<br>Length: 379<br>Identity: 354 / 379 (93.40%)<br>Gaps: 0 / 379 (0.00%)
MUC4-1 Reverse	SW620	282bp	MUC4-1 - SW620 Amplicon Reverse<br>MUC4-1 Isoform<br>10 20 30 40 50 60 70 80 90 100<br>1 CACCACCCTTCTTCTGGTCACCATCCCTTCCTCAGTATCCACAGGTCACGCCACCCCTCTTCCTGTCACCATCCCTTCCTCAGTATCCACAGGTGACACC 100<br>8839 CACCACGCCTCTTCCTGTCACCGACACTTCCTCAGCATCCACAGGTCAGGCCACCCCTCTTCCTGTCACCAGCCTTTCCTCAGTATCCACAGGTGACACC 8938<br>8840 8850 8860 8870 8880 8890 8900 8910 8920 8930<br>MUC4-1 - SW620 Amplicon Reverse<br>MUC4-1 Isoform<br>110 120 130 140 150 160 170 180 190 200<br>101 ACCCCTCTTCCTGTCACCAACCCTTCCTCAGCATCCACAGGTCACGCCACCCCTCTTCCTGTCACCGACACTTCCTCAGCATCCACAGGTCACGCCACCC 200<br>8939 ACCCCTCTTCCTGTCACCGACACTTCCTCAGCATCCACAGGTCACGCCACCTCTCTTCCTGTCACCGACACTTCCTCAGCATCCACAGGTCACGCCACCC 9038<br>8940 8950 8960 8970 8980 8990 9000 9010 9020 9030<br>MUC4-1 - SW620 Amplicon Reverse<br>MUC4-1 Isoform<br>210 220 230 240 250 260<br>201 CTCTTCCTGTCACCAACCCTTCCTCAGCATCCACAGGTCACGCCACCCCTC-TCCTGTCACCGACACT 267<br>9039 CTCTTCCTGACACCGACACTTCCTCAGCATCCACAGGTCACGCCACCCTTCTTCCTGTCACCGACACT 9106<br>9040 9050 9060 9070 9080 9090 9100	Matrix: DNAFULL<br>Gap open penalty: 10.0<br>Gap extend penalty: 1.0<br>Length: 268<br>Identity: 249 / 268 (92.91 %)<br>Gaps: 1 / 268 (0.37 %)
MUC4-1 Reverse	SW620	240bp	MUC4-1 - SW620 Amplicon Reverse<br>MUC4-1 Isoform<br>10 20 30 40 50 60 70 80 90 100<br>1 CCATCCCTTCCTCAGTATCCACAGGTGACACCATGCCTCTTCCTGTCACTAGCCCTTCCTCAGCATCCACAGGTCACGCCACCCCTCTTCCTGTTACCGG 100<br>11,643 CCATCCCTTCCTCAGTATCCACAGGTGACACCATGCCTCTTCCTGTCACTAGCCCTTCCTCAGCATCCACAGGTCACGCCACCCCTCTTCCTGTTACCGG 11,742<br>11650 11660 11670 11680 11690 11700 11710 11720 11730 11740<br>MUC4-1 - SW620 Amplicon Reverse<br>MUC4-1 Isoform<br>110 120 130 140 150 160 170 180 190<br>101 CCTTTCCTCAGCTTCCACAGGTGACACCACCCCTCTTCCTGTCACCGACCCTTCCTCAGCTTCCACAGGTGACACCACCCCTCT--CCGTCACCGACA 196<br>11,743 CCTTTCCTCAGCTTCCACAGGTGACACCACCCCTCTTCCTGTCACCGACACTTCCTCAGCATCCACACGTCACGCCACCCCTCTTCCTGTCACCGACA 11,840<br>11750 11760 11770 11780 11790 11800 11810 11820 11830 11840	Matrix: DNAFULL<br>Gap open penalty: 10.0<br>Gap extend penalty: 1.0<br>Length: 198<br>Identity: 190 / 198 (95.96%)<br>Gaps: 2 / 198 (1.01 %)
