# Supplementary material for: Alternatively Spliced Isoforms of MUC4 and ADAM12 as Biomarkers for Colorectal Cancer Metastasis
Source: J Pers Med. 2023 Jan 10;13(1):135. doi: 10.3390/jpm13010135 (PMC9861497; doi:10.3390/jpm13010135)
Supplement: Supplementary file 1 [file jpm-13-00135-s001.zip › MUC4-All-16-4 Sanger Confidence Score.pdf]

Table S2A: Alignment of MUC4-All Primers using the sample as CCD841, SW480, and SW620 cell lines. Sanger sequencing results were aligned using SnapGene software and NCBI Blast alignment. The confidence scores 98 and 100%.

Gene Name	Cell Line	Amplicon size bp	alignment	Gaps
MUC4-All Forward	CCD841	210bp	MUC4-All - CCD841 Amplicon Forward: 1 CTGGTCTA-AGCCCCCAGAGCGGCTTCACCTGCGTGTCCCCAGTGCAGTAGGGGCTACTGTGACCATGGAGGCCAGTGCCAGCACCTGCC 89<br>MUC4-All Isoforms: 16,040 CTGGTCTACAGCCCCCAGAGCGGCTTCACCTGCGTGTCCCC-GTGCAGTAGGGGCTACTGTGACCATGGAGGCCAGTGCCAGCACCTGCC 16,128<br>MUC4-All - CCD841 Amplicon Forward: 90 CAGTGGGCCCCGCTGCAGCTGTGTGTCCTTCTCCATCTACACGGCCTGGGGCGAGCACTGTGAGCACCTGAGCATGAAACTCGAC 174<br>MUC4-All Isoforms: 16,129 CAGTGGGCCCCGCTGCAGCTGTGTGTCCTTCTCCATCTACACGGCCTGGGGCGAGCACTGTGAGCACCTGAGCATGAAACTCGAC 16,213	Matrix: DNAFULL<br>Gap open penalty: 10.0<br>Gap extend penalty: 1.0<br>Length: 175<br>Identity: 173 / 175 (98.86%)<br>Gaps: 2 / 175 (1.14 %)
MUC4-All Reverse	CCD841	210bp	MUC4-All - CCD841 Amplicon Reverse: 1 GCTTACTTCAGATGCGATGGCTACAAGGGCTACGACCTGGTCTACAGCCCCCAGAGCGGCTTCACCTGCGTGTCCCCATGCAGTAGGGGCTACTGTGACC 100<br>MUC4-All Isoforms: 16,004 GCTTACTTCAGATGCGATGGCTACAAGGGCTACGACCTGGTCTACAGCCCCCAGAGCGGCTTCACCTGCGTGTCCCCGTGCAGTAGGGGCTACTGTGACC 16,103<br>MUC4-All - CCD841 Amplicon Reverse: 101 ATGGAGGCCAGTGCCAGCACCTGCCCAGTGGGCCCCGCTGCAGCTGTGTGTCCTTCTCCATCTACACGGCCT 172<br>MUC4-All Isoforms: 16,104 ATGGAGGCCAGTGCCAGCACCTGCCCAGTGGGCCCCGCTGCAGCTGTGTGTCCTTCTCCATCTACACGGCCT 16,175	Matrix: DNAFULL<br>Gap open penalty: 10.0<br>Gap extend penalty: 1.0<br>Length: 172<br>Identity: 171 / 172 (99.42%)<br>Gaps: 0 / 172 (0.00%)
MUC4-All Forward	SW480	210bp	MUC4-All - SW480 Amplicon Forward: 1 CCCCAGAGCGGCTTCACCTGCGTGTCCCCGTGCAGTAGGGGCTACTGTGACCATGGAGGCCAGTGCCAGCACCTGCCCAGTGGGCCCCGC 90<br>MUC4-All Isoforms: 16,052 CCCCAGAGCGGCTTCACCTGCGTGTCCCCGTGCAGTAGGGGCTACTGTGACCATGGAGGCCAGTGCCAGCACCTGCCCAGTGGGCCCCGC 16,141<br>MUC4-All - SW480 Amplicon Forward: 91 TGCAGCTGTGTGTCCTTCTCCATCTACACGGCCTGGGGCGAGCACTGTGAGCACCTGAGCATGAAACTCGAC 162<br>MUC4-All Isoforms: 16,142 TGCAGCTGTGTGTCCTTCTCCATCTACACGGCCTGGGGCGAGCACTGTGAGCACCTGAGCATGAAACTCGAC 16,213	Matrix: DNAFULL<br>Gap open penalty: 10.0<br>Gap extend penalty: 1.0<br>Length: 162<br>Identity: 162 / 162 (100.00%)<br>Gaps: 0 / 162 (0.00%)
MUC4-All Reverse	SW480	210bp	MUC4-All - SW480 Amplicon Reverse: 1 GCTTACTTCAGATGCGATGGCTACAAGGGCTACGACCTGGTCTACAGCCCCCAGAGCGGCTTCACCTGCGTGTCCCCGTGCAGTAGGGGCTACTGTGACC 100<br>MUC4-All Isoforms: 16,004 GCTTACTTCAGATGCGATGGCTACAAGGGCTACGACCTGGTCTACAGCCCCCAGAGCGGCTTCACCTGCGTGTCCCCGTGCAGTAGGGGCTACTGTGACC 16,103<br>MUC4-All - SW480 Amplicon Reverse: 101 ATGGAGGCCAGTGCCAGCACCTGCCCAGTGGGCCCCGCTGCAGCTGTGTGTCCTTCTCCATCTACACGGCCT 172<br>MUC4-All Isoforms: 16,104 ATGGAGGCCAGTGCCAGCACCTGCCCAGTGGGCCCCGCTGCAGCTGTGTGTCCTTCTCCATCTACACGGCCT 16,175	Matrix: DNAFULL<br>Gap open penalty: 10.0<br>Gap extend penalty: 1.0<br>Length: 172<br>Identity: 172 / 172 (100.00%)<br>Gaps: 0 / 172 (0.00%)
MUC4-All Forward	SW620	210bp	MUC4-All - SW620 Amplicon Forward: 1 CCCCCAGAGCGGCTTCACCTGCGTGTCCCCGTGCAGTAGGGGCTACTGTGACCATGGAGGCCAGTGCCAGCACCTGCCCAGTGGGCCCCGCTGCAGCTGT 100<br>MUC4-All Isoforms: 16,051 CCCCCAGAGCGGCTTCACCTGCGTGTCCCCGTGCAGTAGGGGCTACTGTGACCATGGAGGCCAGTGCCAGCACCTGCCCAGTGGGCCCCGCTGCAGCTGT 16,150<br>MUC4-All - SW620 Amplicon Forward: 101 GTGTCCTTCTCCATCTACACGGCCTGGGGCGAGCACTGTGAGCACCTGAGCATGAAACTCGAC 163<br>MUC4-All Isoforms: 16,151 GTGTCCTTCTCCATCTACACGGCCTGGGGCGAGCACTGTGAGCACCTGAGCATGAAACTCGAC 16,213	Matrix: DNAFULL<br>Gap open penalty: 10.0<br>Gap extend penalty: 1.0<br>Length: 163<br>Identity: 163 / 163 (100.00%)<br>Gaps: 0 / 163 (0.00%)
MUC4-All Reverse	SW620	210bp	MUC4-All - SW620 Amplicon Reverse: 1 GCTTACTTCAGATGCGATGGCTACAAGGGCTACGACCTGGTCTACAGCCCCCAGAGCGGCTTCACCTGCGTGTCCCCGTGCAGTAGGGGCTACTGTGACC 100<br>MUC4-All Isoforms: 16,004 GCTTACTTCAGATGCGATGGCTACAAGGGCTACGACCTGGTCTACAGCCCCCAGAGCGGCTTCACCTGCGTGTCCCCGTGCAGTAGGGGCTACTGTGACC 16,103<br>MUC4-All - SW620 Amplicon Reverse: 101 ATGGAGGCCAGTGCCAGCACCTGCCCAGTGGGCCCCGCTGCAGCTGTGTGTCCTTCTCCATCTACACCGGCCTGG 175<br>MUC4-All Isoforms: 16,104 ATGGAGGCCAGTGCCAGCACCTGCCCAGTGGGCCCCGCTGCAGCTGTGTGTCCTTCTCCATCTACA-CGGCCTGG 16,177	Matrix: DNAFULL<br>Gap open penalty: 10.0<br>Gap extend penalty: 1.0<br>Length: 175<br>Identity: 174 / 175 (99.43%)<br>Gaps: 1 / 175 (0.57%)

Table S2B: Alignment of MUC4-16 Primers using the sample as CCD841, SW480, and SW620 cell lines. Sanger sequencing results were aligned using SnapGene software and NCBI Blast alignment. The confidence scores 99 and 100%.

Gene Name	Cell Line	Amplicon size bp	alignment		Gaps
**MUC4-16 Forward**	**CCD841**	**223bp**	MUC4-16 - CCD841 Amplicon Forward<br>MUC4-16 Isoforms<br><br>MUC4-16 - CCD841 Amplicon Forward<br>MUC4-16 Isoforms	10 20 30 40 50 60 70 80 90 100<br>1 ATAAAAACGGCCACATCCCCATCTTCTTCACCTATGCTGGATAGACACACATCACAACAAATTACAACGGCACCATCAACAAATCATTCAACAATACATT 100<br>1880 ATAAAAACGGCCACATCCCCATCTTCTTCACCTATGCTGGATAGACACACATCCCAACAAATTACAACGGCACCATCAACAAATCATTCAACAATACATT 1979<br>1880 1890 1900 1910 1920 1930 1940 1950 1960 1970<br><br>110 120 130 140 150 160 170<br>101 CCACAAGCACCTCTCCTCAGGAATCACCAGCTGTTTCCCAAAGGGGTCACACTCAAGCCCCGCAGACCACACAAGAATC 179<br>1980 CCACAAGCACCTCTCCTCAGGAATCACCAGCTGTTTCCCAAAGGGGTCACACTCAAGCCCCGCAGACCACACAAGAATC 2058<br>1980 1990 2000 2010 2020 2030 2040 2050	Matrix: DNAFULL<br>Gap open penalty: 10.0<br>Gap extend penalty: 1.0<br><br>Length: 179<br>Identity: 178 / 179 (99.44%)<br>Gaps: 0 / 179 (0.00%)
**MUC4-16 Reverse**	**CCD841**	**223bp**	MUC4-16 - CCD841 Amplicon Reverse<br>MUC4-16 Isoforms<br><br>MUC4-16 - CCD841 Amplicon Reverse<br>MUC4-16 Isoforms	10 20 30 40 50 60 70 80 90 100<br>1 TGGAGAGGCTCTTCTCAGCAGCCCAAGCTACAGTGTGACTCAGATGATAAAAACGGCCACATCCCCATCTTCTTCACCTATGCTGGATAGACACACATCA 100<br>1834 TGGAGAGGCTCTTCTCAGCAGCCCAAGCTACAGTGTGACTCAGATGATAAAAACGGCCACATCCCCATCTTCTTCACCTATGCTGGATAGACACACATCC 1933<br>1840 1850 1860 1870 1880 1890 1900 1910 1920 1930<br><br>110 120 130 140 150 160 170<br>101 CAACAAATTACAACGGCACCATCAACAAATCATTCAACAATACATTCCACAAGCACCTCTCCTCAGGAATCACCAGCTG 179<br>1934 CAACAAATTACAACGGCACCATCAACAAATCATTCAACAATACATTCCACAAGCACCTCTCCTCAGGAATCACCAGCTG 2012<br>1940 1950 1960 1970 1980 1990 2000 2010	Matrix: DNAFULL<br>Gap open penalty: 10.0<br>Gap extend penalty: 1.0<br><br>Length: 179<br>Identity: 178 / 179 (99.44%)<br>Gaps: 0 / 179 (0.00%)
**MUC4-16 Forward**	**SW480**	**223bp**	MUC4-16 - SW480 Amplicon Forward<br>MUC4-16 Isoforms<br><br>MUC4-16 - SW480 Amplicon Forward<br>MUC4-16 Isoforms	10 20 30 40 50 60 70 80 90 100<br>1 AAAACGGCCACATCCCCATCTTCTTCACCTATGCTGGATAGACACACATCACAACAAATTACAACGGCACCATCAACAAATCATTCAACAATACATTCCA 100<br>1883 AAAACGGCCACATCCCCATCTTCTTCACCTATGCTGGATAGACACACATCCCAACAAATTACAACGGCACCATCAACAAATCATTCAACAATACATTCCA 1982<br>1890 1900 1910 1920 1930 1940 1950 1960 1970 1980<br><br>110 120 130 140 150 160 170<br>101 CAAGCACCTCTCCTCAGGAATCACCAGCTGTTTCCCAAAGGGGTCACACTCAAGCCCCGCAGACCACACAAGAAT 175<br>1983 CAAGCACCTCTCCTCAGGAATCACCAGCTGTTTCCCAAAGGGGTCACACTCAAGCCCCGCAGACCACACAAGAAT 2057<br>1990 2000 2010 2020 2030 2040 2050	Matrix: DNAFULL<br>Gap open penalty: 10.0<br>Gap extend penalty: 1.0<br><br>Length: 175<br>Identity: 174 / 175 (99.43%)<br>Gaps: 0 / 175 (0.00%)
**MUC4-16 Reverse**	**SW480**	**223bp**	MUC4-16 - SW480 Amplicon Reverse<br>MUC4-16 Isoforms<br><br>MUC4-16 - SW480 Amplicon Reverse<br>MUC4-16 Isoforms	10 20 30 40 50 60 70 80 90 100<br>1 TGGAGAGGCTCTTCTCAGCAGCCCAAGCTACAGTGTGACTCAGATGATAAAAACGGCCACATCCCCATCTTCTTCACCTATGCTGGATAGACACACATCA 100<br>1834 TGGAGAGGCTCTTCTCAGCAGCCCAAGCTACAGTGTGACTCAGATGATAAAAACGGCCACATCCCCATCTTCTTCACCTATGCTGGATAGACACACATCC 1933<br>1840 1850 1860 1870 1880 1890 1900 1910 1920 1930<br><br>110 120 130 140 150 160<br>101 CAACAAATTACAACGGCACCATCAACAAATCATTCAACAATACATTCCACAAGCACCTCTCCTCAGG 167<br>1934 CAACAAATTACAACGGCACCATCAACAAATCATTCAACAATACATTCCACAAGCACCTCTCCTCAGG 2000<br>1940 1950 1960 1970 1980 1990 2000	Matrix: DNAFULL<br>Gap open penalty: 10.0<br>Gap extend penalty: 1.0<br><br>Length: 167<br>Identity: 166 / 167 (99.40%)<br>Gaps: 0 / 167 (0.00%)
**MUC4-16 Forward**	**SW620**	**223bp**	MUC4-16 - SW620 Amplicon Forward<br>MUC4-16 Isoforms<br><br>MUC4-16 - SW620 Amplicon Forward<br>MUC4-16 Isoforms	10 20 30 40 50 60 70 80 90 100<br>9 TGACTCAGATGATAAAAACGGCCACATCCCCATCTTCTTCACCTATGCTGGATAGACACACATCACAACAAATTACAACGGCACCATCAACAAATCATTC 108<br>1869 TGACTCAGATGATAAAAACGGCCACATCCCCATCTTCTTCACCTATGCTGGATAGACACACATCCCAACAAATTACAACGGCACCATCAACAAATCATTC 1968<br>1870 1880 1890 1900 1910 1920 1930 1940 1950 1960<br><br>110 120 130 140 150 160 170 180 190<br>109 CAAATCATTCAACAATACATTCCACAAGCACCTCTCCTCAGGAATCACCAGCTGTTTCCCAAAGGGGTCACACTCAAGCCCCGCAGACCA 198<br>1969 CAAATCATTCAACAATACATTCCACAAGCACCTCTCCTCAGGAATCACCAGCTGTTTCCCAAAGGGGTCACACTCAAGCCCCGCAGACCA 2058<br>1970 1980 1990 2000 2010 2020 2030 2040 2050	Matrix: DNAFULL<br>Gap open penalty: 10.0<br>Gap extend penalty: 1.0<br><br>Length: 190<br>Identity: 189 / 190 (99.47%)<br>Gaps: 0 / 190 (0.00%)
**MUC4-16 Reverse**	**SW620**	**223bp**	MUC4-16 - SW620 Amplicon Reverse<br>MUC4-16 Isoforms<br><br>MUC4-16 - SW620 Amplicon Reverse<br>MUC4-16 Isoforms	10 20 30 40 50 60 70 80 90 100<br>1 TGGAGAGGCTCTTCTCAGCAGCCCAAGCTACAGTGTGACTCAGATGATAAAAACGGCCACATCCCCATCTTCTTCACCTATGCTGGATAGACACACATCA 100<br>1834 TGGAGAGGCTCTTCTCAGCAGCCCAAGCTACAGTGTGACTCAGATGATAAAAACGGCCACATCCCCATCTTCTTCACCTATGCTGGATAGACACACATCC 1933<br>1840 1850 1860 1870 1880 1890 1900 1910 1920 1930<br><br>110 120 130 140 150 160 170<br>101 CAACAAATTACAACGGCACCATCAACAAATCATTCAACAATACATTCCACAAGCACCTCTCCTCAGGAATCACCAGCTG 179<br>1934 CAACAAATTACAACGGCACCATCAACAAATCATTCAACAATACATTCCACAAGCACCTCTCCTCAGGAATCACCAGCTG 2012<br>1940 1950 1960 1970 1980 1990 2000 2010	Matrix: DNAFULL<br>Gap open penalty: 10.0<br>Gap extend penalty: 1.0<br><br>Length: 179<br>Identity: 178 / 179 (99.44%)<br>Gaps: 0 / 179 (0.00%)

Table S2C: Alignment of MUC4-16 Primers using the sample as CCD841, SW480, and SW620 cell lines. Sanger sequencing results were aligned using SnapGene software and NCBI Blast alignment. The confidence scores 95 and 100%.

Gene Name	Cell Line	Amplicon size bp	alignment		Gaps
MUC4-4 Forward	CCD841	158bp	MUC4-4 - CCD841 Amplicon Forward<br>MUC4-4 Isoform	10 20 30 40 50 60 70 80<br>1 TGAAGGGGGCATGCTGGACGTGGGTCCCCTGGGTGTCCCTGAGCTGCCTGTGTCTCTGCCTCCTTCCGCATGTGGTCCCAGGAATCA 87<br>114 TGAAGGGGGCACGCTGGAGGAGGGTCCCCTGGGTGTCCCTGAGCTGCCTGTGTCTCTGCCTCCTTCCGCATGTGGTCCCAGGAATGA 200<br>120 130 140 150 160 170 180 190 200	Matrix: DNAFULL<br>Gap open penalty: 10.0<br>Gap extend penalty: 1.0<br>Length: 87<br>Identity: 83 / 87 (95.40%)<br>Gaps: 0 / 87 (0.00%)
MUC4-4 Reverse	CCD841	158bp	MUC4-4 - CCD841 Amplicon Reverse<br>MUC4-4 Isoform<br>MUC4-4 - CCD841 Amplicon Reverse<br>MUC4-4 Isoform	10 20 30 40 50 60 70 80 90 100<br>1 GTCTGCTCCTCACACTGCAGCTGCTGGGCCGTGGAGCTTCCCTAGGGAGCCAGGGGGACTTTTGCCGCAGCCATGAAGGGGGCACGCTGGAGGAGGGTCC 100<br>41 GTCTGCTCCTCACACTGCAGCTGCTGGGCCGTGGAGCTTCCCCAGGGAGCCAGGGGGACTTTTGCCGCAGCCATGAAGGGGGCACGCTGGAGGAGGGTCC 140<br>50 60 70 80 90 100 110 120 130 140<br>110 120<br>101 CCTGGGTGTCCCTGAGCTGCCTG 123<br>141 CCTGGGTGTCCCTGAGCTGCCTG 163<br>150 160	Matrix: DNAFULL<br>Gap open penalty: 10.0<br>Gap extend penalty: 1.0<br>Length: 123<br>Identity: 122 / 123 (99.19 %)<br>Gaps: 0 / 123 (0.00%)
MUC4-4 Forward	SW480	158bp	MUC4-4 - SW480 Amplicon Forward<br>MUC4-4 Isoform<br>MUC4-4 - SW480 Amplicon Forward<br>MUC4-4 Isoform	10 20 30 40 50 60 70 80 90 100<br>1 CCCAGGGAGCCAGGGGGACTTTTGCCGCAGCCATGAAGGGGGCACGCTGGAGGAGGGTCCCCTGGGTGTCCCTGAGCTGCCTGTGTCTCTGCCTCCTTCC 100<br>81 CCCAGGGAGCCAGGGGGACTTTTGCCGCAGCCATGAAGGGGGCACGCTGGAGGAGGGTCCCCTGGGTGTCCCTGAGCTGCCTGTGTCTCTGCCTCCTTCC 180<br>90 100 110 120 130 140 150 160 170 180<br>110<br>101 GCATGTGGTCCCAGGAAT 118<br>181 GCATGTGGTCCCAGGAAT 198<br>190	Matrix: DNAFULL<br>Gap open penalty: 10.0<br>Gap extend penalty: 1.0<br>Length: 118<br>Identity: 118 / 118 (100.00%)<br>Gaps: 0 / 118 (0.00%)
MUC4-4 Reverse	SW480	158bp	MUC4-4 - SW480 Amplicon Reverse<br>MUC4-4 Isoform<br>MUC4-4 - SW480 Amplicon Reverse<br>MUC4-4 Isoform	10 20 30 40 50 60 70 80 90 100<br>1 GTCTGCTCCTCACACTGCAGCTGCTGGGCCGTGGAGCTTCCCCAGGGAGCCAGGGGGACTTTTGCCGCAGCCATGAAGGGGGCACGCTGGAGGAGGGTCC 100<br>41 GTCTGCTCCTCACACTGCAGCTGCTGGGCCGTGGAGCTTCCCCAGGGAGCCAGGGGGACTTTTGCCGCAGCCATGAAGGGGGCACGCTGGAGGAGGGTCC 140<br>50 60 70 80 90 100 110 120 130 140<br>110 120<br>101 CCTGGGTGTCCCTGAGCTGC 120<br>141 CCTGGGTGTCCCTGAGCTGC 160<br>150 160	Matrix: DNAFULL<br>Gap open penalty: 10.0<br>Gap extend penalty: 1.0<br>Length: 120<br>Identity: 120 / 120 (100.00%)<br>Gaps: 0 / 120 (0.00%)
MUC4-4 Forward	SW620	158bp	MUC4-4 - SW620 Amplicon Forward<br>MUC4-4 Isoform<br>MUC4-4 - SW620 Amplicon Forward<br>MUC4-4 Isoform	10 20 30 40 50 60 70 80 90 100<br>1 AGGGAGCCAGGGGGACTTTTGCCGCAGCCATGAAGGGGGCACGCTGGAGGAGGGTCCCCTGGGTGTCCCTGAGCTGCCTGTGTCTCTGCCTCCTTCCGCA 100<br>84 AGGGAGCCAGGGGGACTTTTGCCGCAGCCATGAAGGGGGCACGCTGGAGGAGGGTCCCCTGGGTGTCCCTGAGCTGCCTGTGTCTCTGCCTCCTTCCGCA 183<br>90 100 110 120 130 140 150 160 170 180<br>110<br>101 TGTGGTCCCAGGAAT 115<br>184 TGTGGTCCCAGGAAT 198<br>190	Matrix: DNAFULL<br>Gap open penalty: 10.0<br>Gap extend penalty: 1.0<br>Length: 115<br>Identity: 115 / 115 (100.00%)<br>Gaps: 0 / 115 (0.00%)
MUC4-4 Reverse	SW620	158bp	MUC4-4 - SW620 Amplicon Reverse<br>MUC4-4 Isoform<br>MUC4-4 - SW620 Amplicon Reverse<br>MUC4-4 Isoform	10 20 30 40 50 60 70 80 90 100<br>1 GTCTGCTCCTCACACTGCAGCTGCTGGGCCGTGGAGCTTCCCCAGGGAGCCAGGGGGACTTTTGCCGCAGCCATGAAGGGGGCACGCTGGAGGAGGGTCC 100<br>41 GTCTGCTCCTCACACTGCAGCTGCTGGGCCGTGGAGCTTCCCCAGGGAGCCAGGGGGACTTTTGCCGCAGCCATGAAGGGGGCACGCTGGAGGAGGGTCC 140<br>50 60 70 80 90 100 110 120 130 140<br>110 120<br>101 CCTGGGTGTCCCTGAGCTGCC 121<br>141 CCTGGGTGTCCCTGAGCTGCC 161<br>150 160	Matrix: DNAFULL<br>Gap open penalty: 10.0<br>Gap extend penalty: 1.0<br>Length: 121<br>Identity: 121 / 121 (100.00%)<br>Gaps: 0 / 121 (0.00%)
