# Supplementary material for: Alternatively Spliced Isoforms of MUC4 and ADAM12 as Biomarkers for Colorectal Cancer Metastasis
Source: J Pers Med. 2023 Jan 10;13(1):135. doi: 10.3390/jpm13010135 (PMC9861497; doi:10.3390/jpm13010135)
Supplement: Supplementary file 1 [file jpm-13-00135-s001.zip › Supplementary Figure S1&S2.pdf]

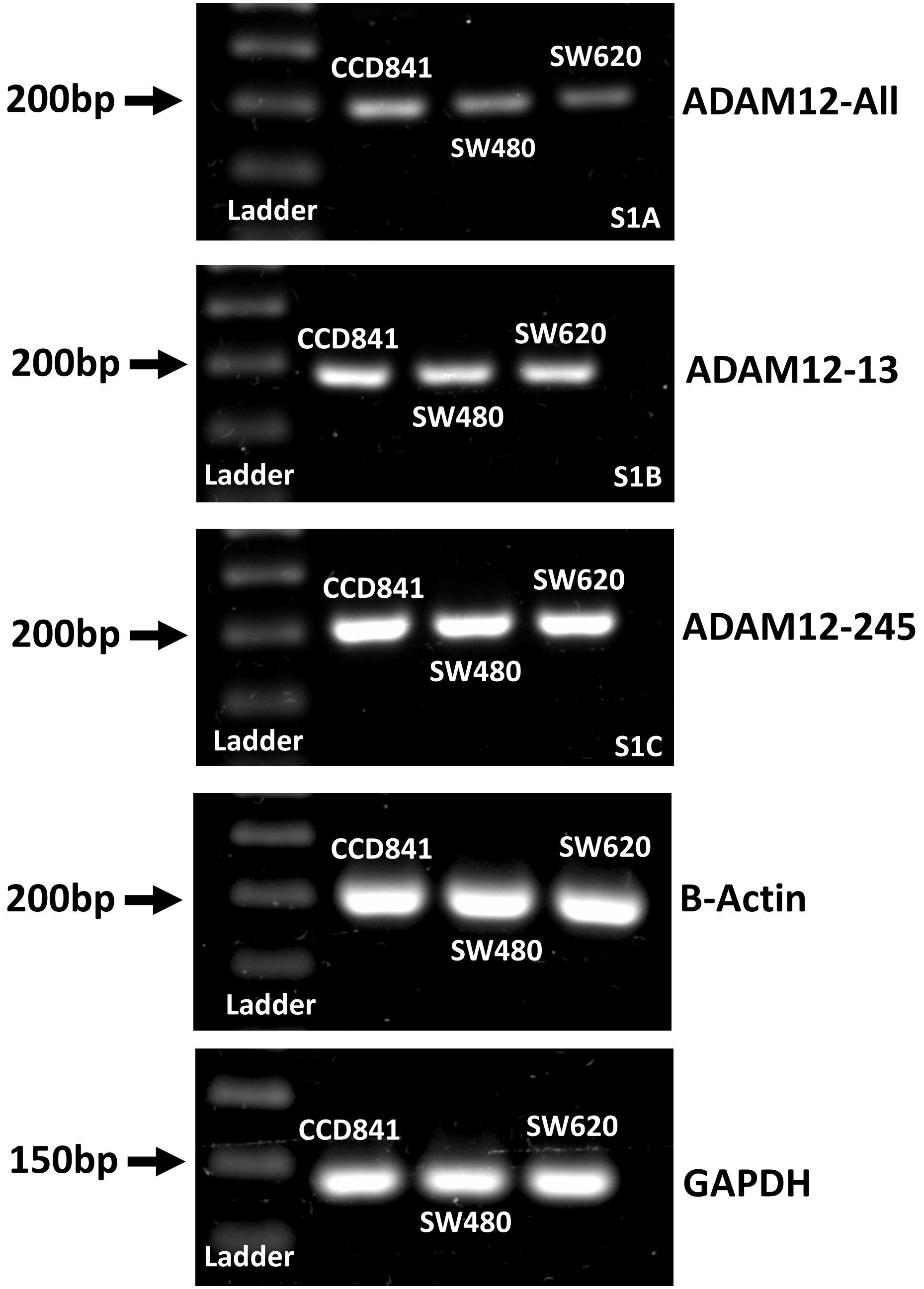

Supplementary Figure S1: EndPoint PCR for the primers ADAM12-All (S1A), ADAM12-13 (S1B), and ADAM12-245 (S1C). The primers B-Actin and GAPDH were expression and loading controls. The templates were the cell lines: CCD841, SW480, and SW620.

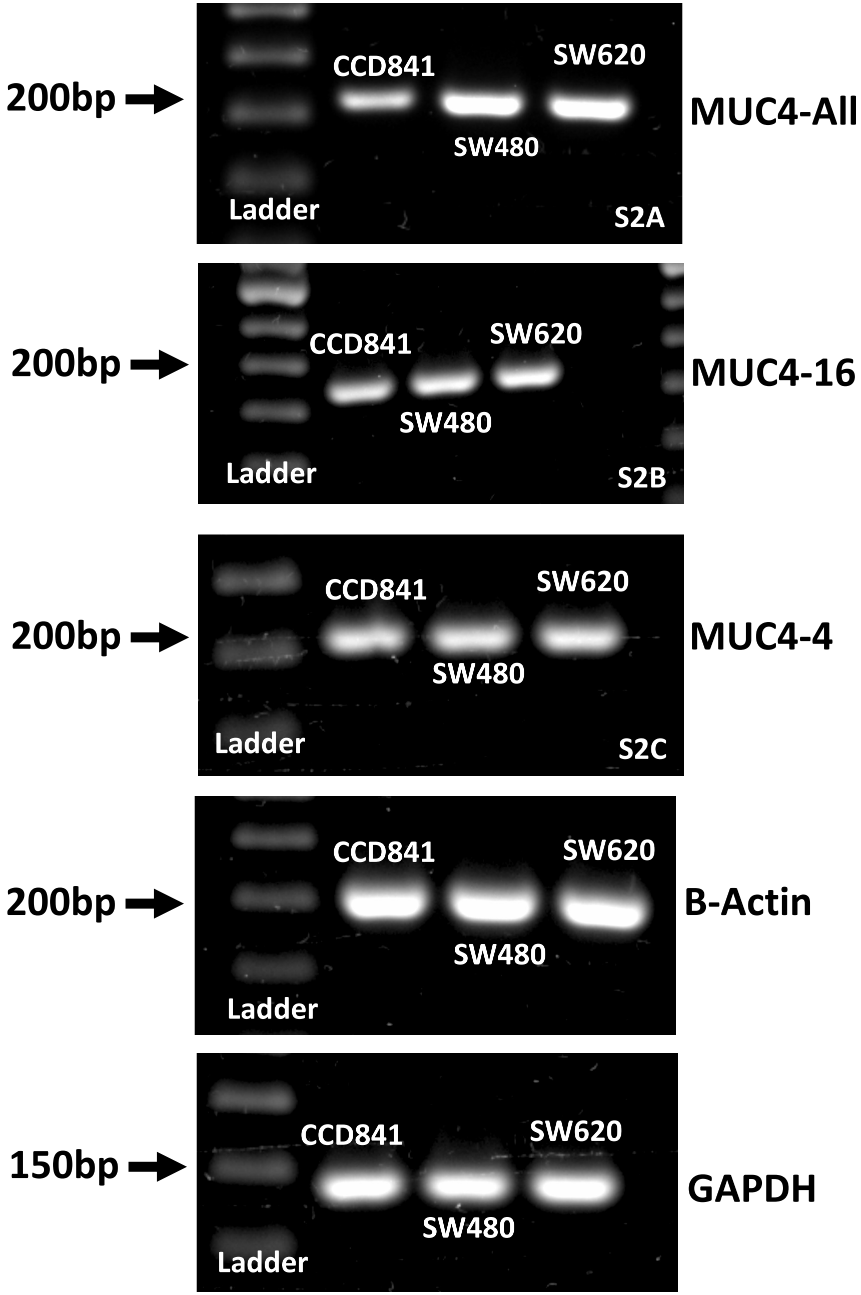

Supplementary Figure S2: EndPoint PCR for the primers MUC4-All (S2A), MUC4-16 (S2B), and MUC4-4 (S2C). The primers B-Actin and GAPDH were expression and loading controls. The templates were the cell lines: CCD841, SW480, and SW620.
