# Supplementary figures and images for: Alternatively Spliced Isoforms of MUC4 and ADAM12 as Biomarkers for Colorectal Cancer Metastasis
Source: J Pers Med. 2023 Jan 10;13(1):135. doi: 10.3390/jpm13010135 (PMC9861497; doi:10.3390/jpm13010135)

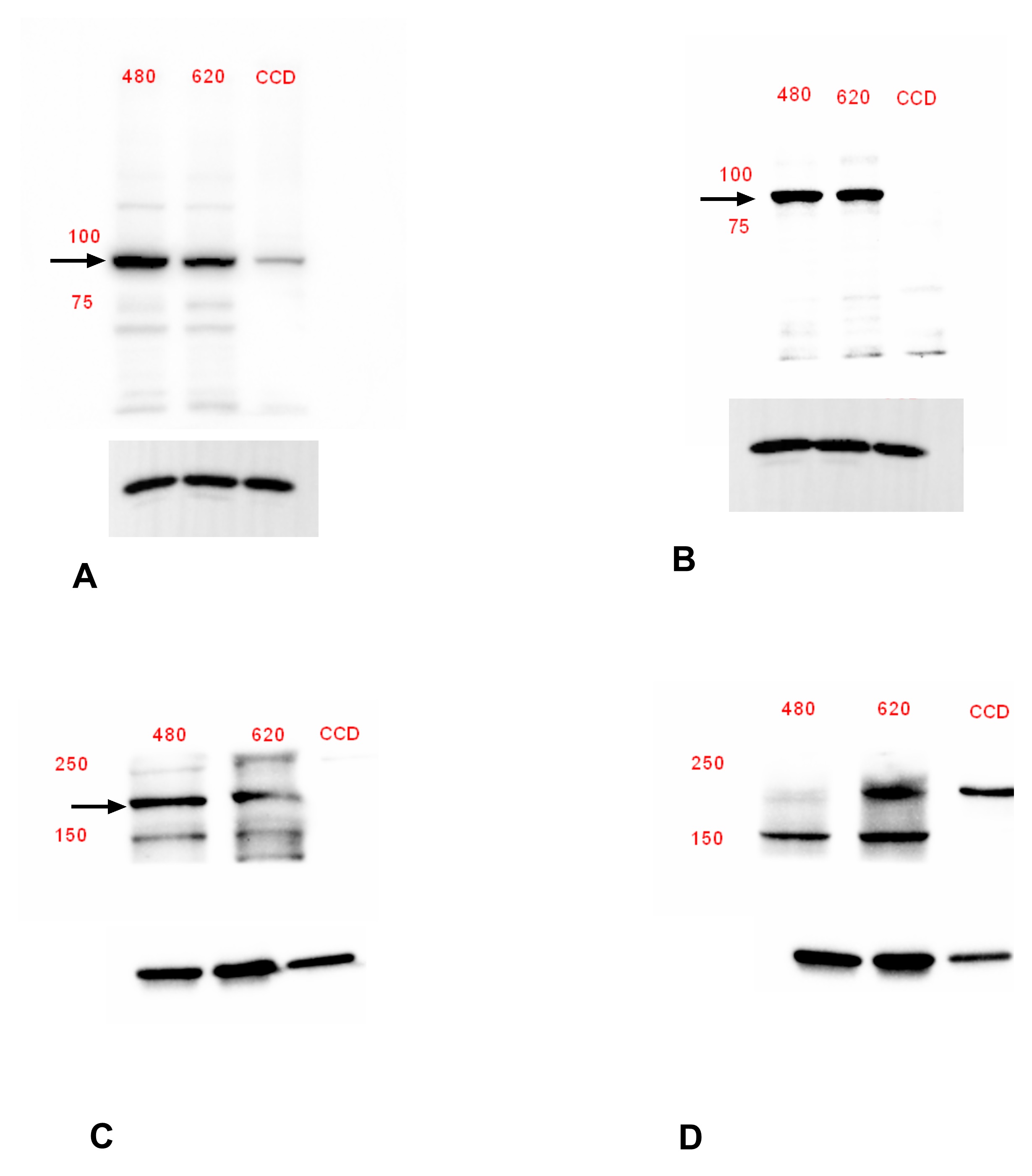

Supplement: Supplementary file 1 [file jpm-13-00135-s001.zip › Supplementary Figure S4.jpg]
